# 360^º^ optoacoustic capsule endoscopy at 50 Hz for esophageal imaging

**DOI:** 10.1016/j.pacs.2022.100333

**Published:** 2022-02-01

**Authors:** Zakiullah Ali, Christian Zakian, Qian Li, Jerome Gloriod, Sophie Crozat, François Bouvet, Guillaume Pierre, Vassilis Sarantos, Massimiliano Di Pietro, Krzysztof Flisikowski, Peter Andersen, Wolfgang Drexler, Vasilis Ntziachristos

**Affiliations:** a Chair of Biological Imaging, Center for Translational Cancer Research (TranslaTUM), School of Medicine, Technical University of Munich, Munich, Germany; b Institute of Biological and Medical Imaging, Helmholtz Zentrum München, Neuherberg, Germany; c Center of Medical Physics and Biomedical Engineering, Medical university of Vienna, Vienna, Austria; d Statice, Besancon, France; e Sonaxis S.A., Besancon, France; f RayFos: Scientific Software, Basingstoke, United Kingdom; g MRC Cancer Unit, University of Cambridge, Cambridge, United Kingdom; h Chair of Livestock Biotechnology, School of Life Science, Technical University of Munich, Freising, Germany; i Department of Health Technology, Technical University of Denmark, Lyngby, Denmark

**Keywords:** **OA**, Optoacoustics, **RPM**, Revolutions per minute, **GI**, Gastrointestinal, **WLSE**, White light Surveillance Endoscopy, Optoacoustic endoscopy, Ultrasound transducer, 360° field of view, Fast imaging, Distal scanning, Slip rings, Photoacoustics

## Abstract

Gastrointestinal (GI) endoscopy is a common medical diagnostic procedure used for esophageal cancer detection. Current emerging capsule optoacoustic endoscopes, however, suffer from low pulse repetition rates and slow scanning units limit attainable imaging frame rates. Consequently, motion artifacts result in inaccurate spatial mapping and misinterpretation of data. To overcome these limitations, we report a 360^º^, 50 Hz frame rate, distal scanning capsule optoacoustic endoscope. The translational capability of the instrument for human GI tract imaging was characterized with an Archimedean spiral phantom consisting of twelve 100 µm sutures, a stainless steel mesh with a pitch of 3 mm and an *ex vivo* pig esophagus sample. We estimated an imaging penetration depth of ~0.84 mm in vivo by immersing the mesh phantom in intralipid solution to simulate light scattering in human esophageal tissue and validated our findings *ex vivo* using pig esophagus. This proof-of-concept study demonstrates the translational potential of the proposed video-rate endoscope for human GI tract imaging.

## 1 Introduction

Having caused a total of 544,076 deaths in 2020, esophageal cancer is the sixth deadliest cancer and responsible for 1 in every 19 cancer-related deaths worldwide [1]. The prognosis of patients with advanced esophageal cancer is poor, with survival rates less than 20% after five years. However, early detection of the disease can allow for curative treatment and potential survival rates exceeding 80% [2]. At present, esophageal cancer is detected by white light surveillance endoscopy (WLSE), where the lumen is illuminated with a standard color camera that is designed to create images that replicate human vision, sensitive to only red, green, blue wavelengths (RGB) for accurate colour representation. Unfortunately, changes in contrast arising from low-grade dysplasia or early cancerous lesions are often imperceptible when imaging the esophagus in vivo, even with high definition WLSE. Therefore, arbitrary quadratic biopsies for histological examination are required to increase the rate of lesion detection [3], [4], [5]. Recently, a clinically translatable hyperspectral endoscope (HySE) system was developed to improve contrast for in vivo gastrointestinal (GI) tract imaging and features excellent spatial, spectral, and temporal resolution, as well as high color fidelity [6]. HySE applications include quantification of blood oxygenation levels and differentiation of spectral profiles from normal to pathological ex vivo human tissues. However, HySE does not resolve depth information and image feature extraction is still challenged in homogenous or low contrast situations. As an alternative, ultrasound endoscopy is employed to obtain structural depth information within the esophageal wall for cancer staging [7]. Despite ultrasound and WLSE’s broad use, the compromises of these technologies include limitations in contrast and depth. WLSE only allows visual inspection of the esophageal surface, ultrasound endoscopy offers no molecular contrast and has low resolution within the upper mucosal layers, and biopsies can often miss early stages of the pathology [7]. As a consequence, depth-resolved information across the esophageal layers is necessary to increase the early detection rate of esophageal cancer and accurately classify the disease’s stage. The average healthy esophagus wall is 5.26 mm thick for males and 4.34 mm thick for females [8] and is highly vascularized with vessels ranging from tens to several hundreds of micrometers in size within the mucosa [9].

Optoacoustic (OA) endoscopy is a potentially promising technique for esophageal cancer screening as it can deliver three dimensional, label-free, structural and functional imaging in vivo, offering high resolution molecular imaging through several millimeters of tissue [10], [11]. Vascular remodeling and angiogenesis are common landmarks of tumor lesions [12] and the spectral absorption properties of hemoglobin in red blood cells offer an intrinsic optical molecular absorption contrast mechanism to map the vascular network using optoacoustics [13]. The imaging modality yields sub-surface visualization useful for identifying specific alterations of microvascular patterns for the detection of dysplasia and cancer [14]. Visualization of the vascular network through tissue layers can support assessment of tumor invasion and accurate staging of esophageal cancer [15], particularly in early phases of the disease where optimized treatment plans can lead to improved prognoses in the clinic.

OA imaging is based on ultrasound detection induced by light absorption and thermoelastic expansion of molecules within biological tissues, thereby offering high molecular specificity and rich contrast at greater depths than purely optical imaging [16]. These features render OA endoscopy a potentially powerful modality for evaluating and staging malignant tissue within the gastrointestinal (GI) tract. Current OA endoscopes however suffer from low imaging speeds, incomplete fields of view and poor signal-to-noise-ratios (SNR). In order to deliver performance relevant for medical use, imaging the GI tract with optoacoustic endoscopy requires: a) high imaging frame rates to avoid motion artefacts, b) large field of views to expedite the screening procedure, c) careful design of broadband transducers with a miniaturized form factor to be inserted through hollow bodily cavities, and d) appropriate probe geometry to ensure mechanical stability and for maintaining optimal contact between the endoscope and the esophageal circumference for acoustic index matching.

Existing implementations of OA endoscopy include a 4 Hz-distal rotation endoscope which was employed in simultaneous optoacoustic and ultrasonic endoscopy for imaging internal organs of a rabbit [17]. However, the device has a limited field of view due to driving cables crossing over the imaging plane, with the use of reflective ultrasound elements refracting sound and leading to distortions and signal attenuation. Another group showed a proximal scanning catheter with the ability to perform full field of view optoacoustic and ultrasonic dual modality imaging of colorectal tissue of a Sprague Dawley rat [18]. However, this system is limited to a slow frame rate of 5 Hz due to low laser repetition rate and the rotation speed of the rotary joint. A fast endoscope was demonstrated with proximal rotation reaching 50 Hz with a 300 kHz repetition rate laser and high speed rotatory joint to image the rectum wall of a Sprague Dawley rat [19]. This design suffers from poor lateral resolution due to the use of an unfocused acoustic detector and loss of SNR due to lower light transmission efficiency through the fiber optic rotatory joint. The drop in SNR necessitated the use of a 15 mJ/cm^2^ fluence to obtain images, significantly exceeding the maximum permissible exposure limit (MPE) at high repetition rates (see discussion). Furthermore, proximal rotation via torque coils are problematic due to uncontrolled coil friction causing heat, limiting the rotation speed, and affecting scanning uniformity during operation. Loss of scanning uniformity results in loss of image formation accuracy. The above-mentioned configurations [17], [18], [19] focused on OA esophageal endoscopes for small animals, aimed at further miniaturization of the probe for insertion into the working channel of white endoscopes. Nevertheless, these solutions exhibit poor OA coupling against the wall due to the dimension of the human esophageal lumen and long propagation distances to the lumen surface, leading to an overall loss of SNR. A side looking optoacoustic capsule endoscope designed to be in direct contact with the esophagus wall could improve the OA coupling and OA sensitivity by placing the sensor closer to the interrogated tissue surface. A prototype capsule-shaped acoustic resolution optoacoustic probe was shown to to characterize the intestinal structures of Crohn’s disease in a rabbit [20]. However, the imaging speed was limited due to a slow laser pulse repetition rate of 10 Hz and a low SNR, necessitating the signal to be averaged 30 times, slowing acquisition times considerably. Our group [21] has recently demonstrated a side looking capsule endoscope optoacoustic imaging prototype with a 360° field of view; however, the imaging speed that was limited by a laser repetition rate of 2 kHz and the proximal rotational scanning unit rate of 2.5 Hz.

Clinical translation of current OA capsule endoscopes are limited by their slow scanning speeds. Our approach reported herein instead offers video rate (50 Hz), 360-degrees, side-looking OA signal acquisition by implementing distal rotation coupling of the acoustic sensor and laser illumination, paving the way towards clinical translational of OA capsule endoscopy of the GI track. We characterized the endoscope’s lateral resolution by imaging a phantom consisting of twelve 100 µm sutures arranged in an Archimedean spiral with a pitch of 3 mm, and demonstrated a volumetric pullback spiral scan over 1790 mm^3^ at 0.24 Hz. To simulate scattering of light in esophageal tissue, the acoustic coupling medium of water was replaced with various concentrations of an intralipid solution to determine an estimated penetration depth of ~0.84 mm in the human esophagus. To validate the estimated penetrated depth, we imaged an *ex vivo* pig esophagus. Our results demonstrate proof-of-principle translational potential of optoacoustic endoscopy for delivering video-rate 3D functional information of the human esophagus.

## 2 Material and methods

**Fig. 1:**
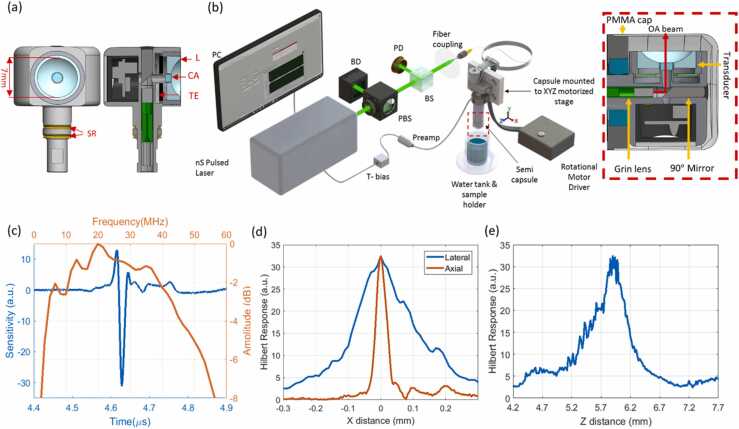
Endoscopic transducer characterization (a) Cross-sectional and en-face view of the endoscopic transducer with a central aperture. TE: Transducer element, EC: Electrical Connector, L: Acoustic Lens, CA: Central Aperture Window, SR: Slip rings. (b) Benchtop optoacoustic endoscopy experimental setup. PBS: polarizing beam splitter, BS: beam splitter, BD: beam dump, PD: photodiode. (c) Optoacoustic transducer temporal and frequency response. (d) Axial and lateral Hilbert transducer resolutions (e) Hilbert depth-of-focus response.

The benchtop optoacoustic endoscope unit as illustrated in Fig. 1 consists of a ns-pulse width, 532 nm Q-Switched DPSS laser with an operating repetition rate in the range of 1–100 kHz (ONDA, Bright Solutions, Italy). The energy per pulse was regulated by means of a polarizing beam splitter and a beam dump. A 90:10 beam splitter was employed to divert 10% of the light towards a photodetector for triggering. The remaining 90% of light was coupled into a fiber to guide the optical beam to the optoacoustic semi capsule endoscope.

The proposed 16 mm diameter semi-capsule prototype consists of a miniature motor with an ultrafine hollow shaft (Maxon Group, Switzerland) to allow for coaxial low–beam divergence optoacoustic illumination with a custom designed GRIN lens mounted to the distal end of the multimode optical fiber with a core diameter of 105 µm and numerical aperture of 0.2 (GRINTECH, Germany). The detector features a 28 MHz-central frequency, side-looking, planar lithium niobate (LiNbO_3_) active element transducer with a 6 mm-fused silica acoustic lens, and a central aperture of 2 mm in diameter to allow coaxial illumination (SONAXIS, France). To facilitate side illumination, the transducer is integrates a 90° silver coated optical reflector with > 95% reflectivity at 532 nm, surface flatness of λ/4, and surface quality of 60–40 scratch-dig. Furthermore, a sealing window manufactured with PMMA altuglas SG7 with a thickness of 1 mm was installed at the central aperture of the detector. The optical beam was a low divergence beam with a half divergence angle of 3.9° and focal length of 7.4 mm along the air and water optical path length inside the capsule. Full 360° unobstructed field of view is achieved by implementing customized low friction miniature gold slip rings (STATICE, France) to enable electrical coupling of high frequency ultrasound signals, thereby avoiding obstruction from driving cables over the field of view. The capsule is encapsulated in water with a cylindrical PMMA capsule lid featuring a thin fiducial marker used for synchronization and interpolation of high frame rate B scans.

In order to drive the rotational motor, an ESCON module 24/2, 4-Q servo driver was operated using ESCON studio via PC. The optoacoustic signals measured during rotational scanning were pre-amplified externally with an 30 dB amplifier (Sonaxis SA, France) and connected to a T-bias (ZFBT-4R2GW+, Mini-Circuits) prior to data acquisition at a sampling rate of 500 MHz. The T-bias is used to provide a DC voltage supply to the pre-amplifier circuit while remaining decoupled from the radiofrequency (RF) line of the transducer, hence voltage fluctuations from the DC voltage source do not affect the RF signal. The semi capsule prototype was mounted to an XYZ linear motorized stage (MTS50-Z8 in 3-axis XYZ Configuration Thorlabs) to allow for controlled repositioning and performed 3D Helical pull-back scans. The optoacoustic data was acquired by a ATS9373 DAQ card (AlazarTech, Canada) synchronized to the board’s external trigger port via the photodetector signal. Signal processing was controlled by LabVIEW and data analyzed in MATLAB.

The endoscopic transducer was characterized by linear raster scanning the semi capsule against a 20 µm suture in the YZ plane by shifting the transducer away from the acoustic source generated via the suture (y steps = 10 µm, z steps = 5 µm) to determine the temporal/frequency response at focus, the axial/lateral resolutions, and depth-of-focus (See supplementary for further information).

**Fig. 2:**
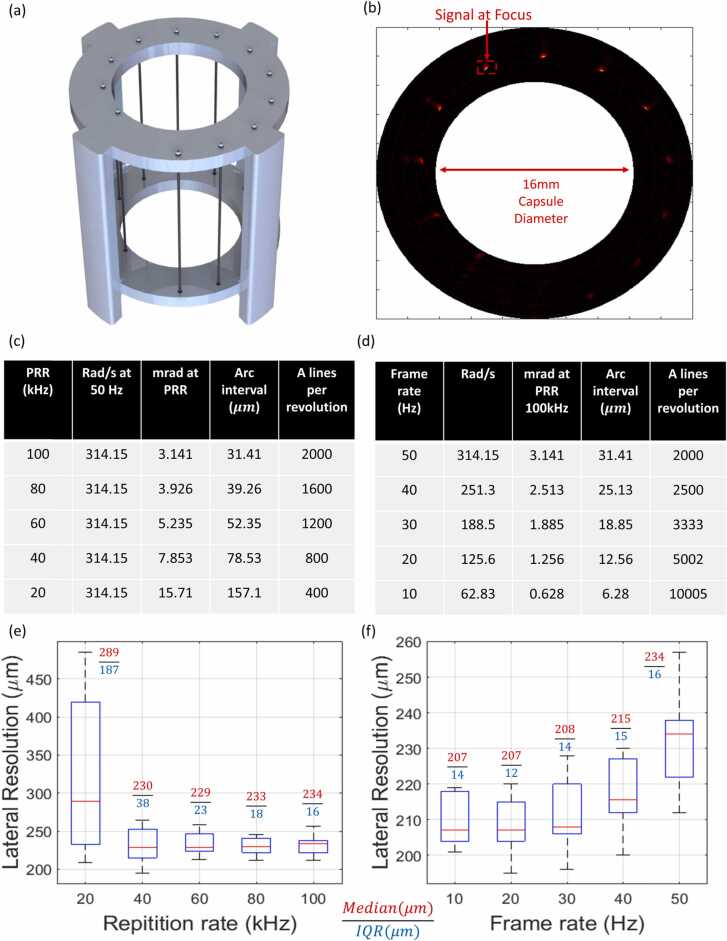
Endoscope performance at 50 Hz B-scan frame rate (a) Twelve sutures with 100 µm thickness arranged in an Archimedean spiral phantom with a pitch of 3 mm. (b) 360º B scan image of phantom acquired at 50 Hz and 100 kHz pulse repetition rate. (c) Table listing the number of A lines per revolution and arc sampling interval at the transducer focal plane as a function of pulse repetition rate at a fixed frame rate of 50 Hz. (d) Table listing the number of A lines per revolution and arc sampling interval at the transducer focal plane as a function of frame rate at a fixed pulse repetition rate of 100 kHz (e) Lateral resolution measured at transducer focus at 50 Hz frame rate as a function of laser pulse repetition rate under the same experimental configurations listed in table (c). (f) Lateral resolution measured at transducer focus at 100 kHz laser pulse repetition rate as a function of B scan frame rate under the same experimental configurations listed in table (d).

To demonstrate 50 Hz B-scan frame rate with 100 kHz pulse repetition rate (31.4 µm focal plane lateral sampling interval), a phantom comprising twelve 100 µm sutures arranged in an Archimedean spiral with a pitch of 3 mm as depicted in Fig. 2(a) was imaged. The phantom was designed to ensure that one of the sutures lies within the focal plane of the transducer to determine the influence of laser pulse repetition rate and B-scan frame rate on the lateral resolution at focus.

**Fig. 3:**
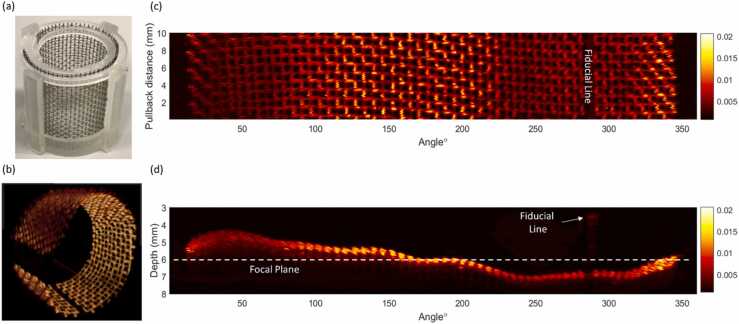
Helical volumetric Cscan over 10 mm longitudinal pullback distance at 50 Hz B-scan rate and 100 kHz repetition rate (a) Aluminum phantom mesh mounted in an Archimedean spiral with a pitch of 3 mm. (b) 3D polar projection of Helical optoacoustic scan generated on Amira (c) 2D maximum intensity projection of Helical scan depicted in Cartesian coordinates. (d) 2D Axial maximum intensity projection of Helical scan depicted in Cartesian coordinates.

**Fig. 4:**
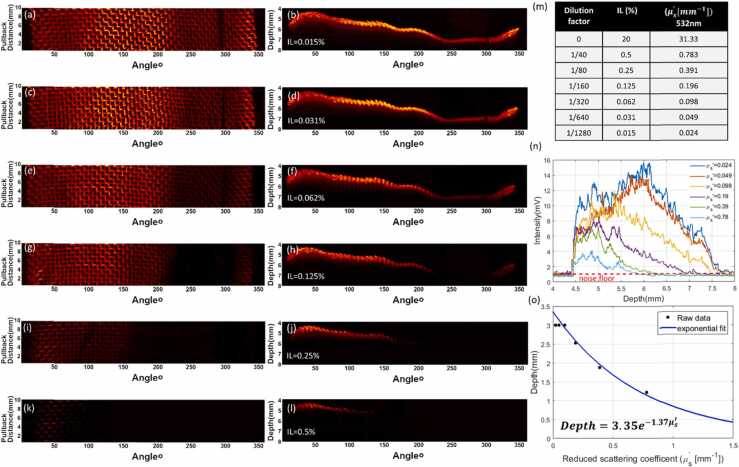
360º helical volumetric scanning over a 10 mm longitudinal pullback distance with intralipid solution**.** (a,b) 2D top and side maximum intensity projections, respectively, with 0.015% intralipid solution. The corresponding figure pairs (c,d), (e,f), (g,h),(i,j), (k,l) represent the 2D projections with 0.031%, 0.062%,0.125%,0.25%,0.5% intralipid concentration, respectively. (m) Table listing the corresponding reduced scattering coefficient $\mu_{s}^{\prime }$ at each IL concentration at 532 nm (n) The maximum intensity profile as a function of depth for all scattering coefficients (o) The relationship between depth and reduced scattering coefficient.

A 3D helical pull back optoacoustic scan over 10 mm of a stainless steel mesh arranged in an Archimedean spiral with a pitch of 3 mm (Fig. 3a) was performed at 50 Hz B-scan rate and 100 kHz repetition rate with a pullback speed of 2.4 mm/s over 4.16 s acquiring a volume of 1700 × 2000×208 (ρ,φ,z) voxels (or 5 mm×360º x 10 mm or 1790 mm^3^). This experiment was repeated by replenishing the acoustic coupling medium with controlled titration concentrations of intralipid (IL) solution to simulate the scattering of light during the interrogation of human esophageal tissue. A 20% IL solution was diluted to six test IL concentrations including 0.015%, 0.031%, 0.062%, 0.125%, 0.25% and 0.5%. The main molecular absorber in mucosa at 532 nm is hemoglobin, which is in circulation in blood vessels (simulated by the mesh) whereas the connective tissue in mucosa acts primarily as a scattering medium at this wavelength (simulated by the IL). The detection depth limit was defined by the deepest position where the signal is greater than the system noise level of 1 mV, which is SNR= 1 for the intralipid mesh experiment shown in Fig. 4(o).

The same scanning configuration was also performed on an *ex vivo* pig esophageal sample mounted inside a 50 mL-Falcon tube to demonstrate translational potential. In an attempt to preserve the vascular network in the esophagus during sample extracting, a heated blade was used to cut and seal each end of the esophagus. The pulse laser energy was measured at the capsule surface during all imaging and was maintained at ~12 µJ per pulse. Energy measurements were initially made using an Ophir energy meter (model PE10-C) which can operate up to a pulse repetition rate of 25 kHz. The OA signal strength generated by a reference marker was used as a surrogate monitor of the pulse energy for repetition rates beyond the limit of the energy meter to keep the pulse energy ~12 µJ per pulse.

## 3 Results

### 3.1 Endoscopic optoacoustic transducer characterization

The structure and setup of the proposed endoscopic transducer is shown in Fig. 1a and b and described in the Methods section. The detector characterization procedure revealed the spatial features of the transducer. The sensor exhibited a broadband detection performance with a central frequency of 28 MHz and a − 6 dB bandwidth of 50 MHz as depicted in Fig. 1(c). The temporal response indicated a transducer focal length of 6 mm. The axial and lateral resolutions were measured as 35 µm and 200 µm, respectively, as shown in Fig. 1(d). A 700 µm depth-of-focus was measured from the Hilbert plot shown in Fig. 1(e).

### 3.2 OA endoscopic imaging of a suture phantom at 50 Hz B-scan frame rate

To characterize endoscope’s lateral resolution performance as a function of laser pulse repetition rate and scanning speed, we first imaged a phantom comprised of twelve 100 µm sutures arranged in an Archimedean spiral with a pitch of 3 mm (Fig. 2a). Fig. 2(b) depicts a B scan acquired at 50 Hz with a pulse repetition rate of 100 kHz. The B scan highlights the near-field, focus, and far-field response of the transducer, with the sensor sensitivity reaching its maximum value at focus which is located 2 mm away from the capsule surface.

To assess the influence of the arc sampling interval and scanning speed on the lateral resolution at focus, we acquired continuous scans at the maximum frame rate (50 Hz) with different laser pulse repetition rates (20–100 kHz), followed by scans at the maximum repetition rate (100 kHz) with different frame rates (10–50 Hz). For each case we acquired a set of 10 continuous revolutions and calculated the lateral resolution for each rotation scan. The median and interquartile ranges (IQR) of the measurements were used as indicators of resolution and variability for each condition.

Table (c) in Fig. 2 shows the arc sampling intervals and number of A lines obtained per revolution at a constant B frame rate of 50 Hz for pulse repetition rates ranging between 20 kHz and 100 kHz. The median lateral resolution between repetition rates 40–100 kHz is approximately the same, varying between 229 and 234 µm. However, at 20 kHz the median lateral resolution degrades to a value of 289 µm. The reduced median lateral resolution of 289 µm at 20 kHz laser pulse rate is attributed to a low lateral arc sampling interval of 157 µm at the focal plane. This case yielded values ranging between 209 µm and 485 µm, which is expected due to the violation of a Nyquist sampling criteria for sampling at a minimum of double the resolvable lateral resolution. For the proposed transducer this would correspond to an approximately 100 µm arc sampling interval. Additionally, as the repetition rate is increased from 20 to 100 kHz, the variability between revolutions decreases due to an increased angular sample resolution per B frame; and we therefore observed a decrease in the IQR from 187 µm to 16 µm.

Table (d) in Fig. 2 shows the arc sampling intervals and number of A lines obtained per revolution at a constant laser pulse repetition rate of 100 kHz for B frame scanning rates ranging between 10 Hz and 50 Hz. The resulting lateral resolution distribution measurements are plotted in Fig. 2(f). Here, the median lateral resolution improved from 234 µm (50 Hz) to 207 µm (10 Hz), almost matching the lateral resolution of the transducer of 200 µm as characterized in Fig. 1. Although improvement in arc sampling interval is always obtained when reducing frame rates, in this instance no significant improvement in the IQR, fluctuating between 12 and 16 µm, was observed over the 10–50 Hz test B frame rates.

Overall, by reducing the B scan frame rate and using a higher laser pulse repetition rate, arc sampling intervals are reduced during measurement which, however, results in an increased number of A scans per revolution, impacting file size and memory management. Nevertheless, our results show arc sampling intervals below 18.8 µm obtained using a 30 Hz scan rate and 100 kHz repetition rate, indicating no significant improvement in the median value of the lateral resolution corresponding to one tenth of the transducer lateral resolution.

### 3.3 Volumetric OA endoscopy imaging of a mesh phantom at 50 Hz B-scan frame rate

We next set out to demonstrate the volumetric imaging capability of the endoscope. We performed a Cscan of a stainless steel mesh at 50 Hz B-scan rate arranged in an Archimedean spiral set at a pitch of 3 mm (Fig. 3a) which served as a phantom sample, with the minimum diameter of 16 mm and the maximum of 22 mm falling within the range of a human esophagus diameter [22]. Fig. 3(b) depicts the 3D projection of the helical scan at 50 Hz and 100 kHz repetition rate pulled back over 10 mm at 2.4 mm/s. The full scan was acquired in 4.16 s with an arc sampling resolution of 31.4 µm at focus (r = 6 mm), an axial sampling resolution of 3 µm (Fs = 500 MHz), and a helical pitch of 48 µm. Fig. 3(c) and (d) illustrate the 2D maximum top and axial intensity projections generated from the helical scan, respectively, with the fiducial marker and focal plane highlighted. The maximum intensity projections indicate that the intensity values are highest at focus, as expected for a focus transducer; however, the mesh appears to be distinguishable over the entire field of view across the total depth of 3 mm.

### 3.4 Volumetric OA endoscopic imaging of a mesh phantom immersed in intralipid solution to mimic tissue scattering at 50 Hz B-scan frame rate

To assess the imaging penetration depth of the endoscope under light scattering conditions mimicking those of the human esophagus, we embedded the mesh in an intralipid (IL) solution at different concentrations. The maximum intensity projections from the top and side views of the volumetric scans were obtained and the fiducial marker masked out to avoid signal saturation and to restrict the analysis to intensities within the dynamic range from the mesh in the scattering media.

At 0.015% IL (Fig. 4a and b), the maximum signal strength was 15.9 mV and the entire mesh was visible over the full 3 mm depth of the mesh. At 0.031% IL (Fig. 4c and d), the maximum signal strength was reduced to 14 mV, but the entire mesh remained observable over the full depth. At 0.062% IL (Fig. 4e and f), the maximum signal strength dropped to 11.9 mV, with the mesh still perceivable over the full 3 mm depth, but losing definition of the grid pattern in the far field. At 0.125% IL (Fig. 4g and h), the maximum signal strength was 8.1 mV and the mesh in the far field was no longer observable, hence limiting the imaging depth to 2.53 mm. At 0.250% IL (Fig. 4i and j), the maximum signal strength and depth were further reduced to 7.5 mV and the mesh was only observable in the near field, limiting the imaging depth to 1.88 mm. Finally, at 0.50% IL (Fig. 4k and l), the maximum signal strength reduced to 4.05 mV, the grid pattern of the mesh was no longer defined and the imaging depth was limited to 1.22 mm.

Light scattering and diffusion affects the amount of light available for OA generation from tissue. To estimate the expected imaging depth in the human esophagus, we calculated the reduced scattering coefficient, $\mu_{s}^{\prime }$, at 532 nm for the IL solutions in our experiments based on previously reported values at 20% IL concentration [23]. Table (m) in Fig. 4 shows the $\mu_{s}^{\prime }$ values for each IL concentration (0.015–0.5%).

Fig. 4(n) shows the maximum intensity values for the volumetric mesh images as a function of depth. We determined the position at which the signal intensity drops below the noise level, which was identified from the average intensity value at depths between 4 mm and 4.4 mm (Fig. 4n), where no part of the mesh is present in our sample, yielding a background noise level of ~1 mV. We therefore used 1 mV-noise level to estimate the imaging depth achievable for each scattering conditions using our OA endoscopic configuration. Interestingly, we observed that the intensity depth profiles agree with the response of our focused detector (see Fig. 1e) for the low scattering cases with $\mu_{s}^{\prime }$ = 0.024 and $\mu_{s}^{\prime }$ = 0.049. Here, the peak intensity is seen at 6 mm, corresponding to the focal point of the transducer. For $\mu_{s}^{\prime }$ at and above 0.098, the peak intensity shifts towards the near field of the transducer as expected from the reduced light penetration with increased scattering. This shift suggests that the focal distance of the transducer could be tuned close to the surface of the sample, matching the highest sensitivity of the transducer to the attainable light penetration available for OA signal generation, therefore increasing the SNR and thereby penetration depth under high scattering media conditions.

Fig. 4(o) illustrates the relationship between $\mu_{s}^{\prime }$ and penetration depth. The data is fit to an exponential equation of the form 3.35exp(−1.37$\mu_{s}^{\prime }$). Healthy human esophagus has been previously reported with $\mu_{s}^{\prime }$=$1.01\pm 0.14mm^{-1}$ at 526 nm [24]. We chose a scattering coefficient reported for superficial mucosal layers (highlighted in Table S1) rather than a fully perfused esophagus, as our model splits the contribution of blood absorption using the mesh inserted in the phantom. Assuming an insignificant change in $\mu_{s}^{\prime }$ from 526 nm to 532 nm, and following our derived expression for the penetration depth and $\mu_{s}^{\prime }$, we predict an imaging penetration depth in the human esophagus of ~0.84 mm exceeding the MPE by a factor of 4 at a 100 kHz-pulse repetition rate and 50 Hz-B-scan frame rate (see Supplementary).

### 3.5 Volumetric OA endoscopic imaging of an ex vivo pig esophagus at 50 Hz B-scan frame rate

**Fig. 5:**
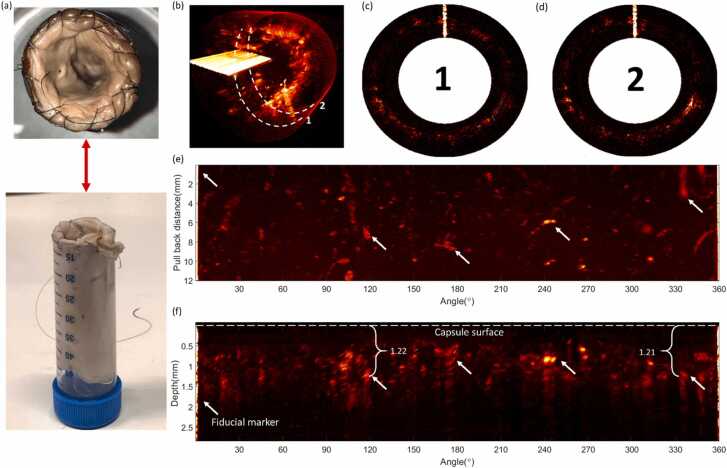
360º helical volumetric scanning of an *ex vivo* female pig esophagus over a 12 mm longitudinal pullback distance. (a) *Ex vivo* pig esophagus sample mounted on a 50 mL-Falcon tube for support (b) 3D volumetric render of helical scan (c,d) B scans taken at unique positions within the helical scan. Maximum intensity projection for (e) enface and (f) side views.

To validate our phantom model and demonstrate translation potential of our endoscope, we imaged an *ex vivo* female pig esophagus (Fig. 5a) mounted inside a 50 mL falcon tube. Fig. 5(b) illustrates the 3D volume of the acquired pullback scan at 50 Hz B scan rate and 100 kHz over a pullback distance of 12 mm at 2.4 mm/s. The full scan was acquired in 5 s with an arc sampling resolution of 31.4 µm at focus, an axial sampling resolution of 3 µm, and a helical pitch of 48 µm. Fig. 5(c) and (d) depict B scans taken from two arbitrary positions from the helical pull back scan demonstrating the ability to acquire data over a 360° field of view. Fig. 5(e) and (f) illustrate the 2D enface and axial maximum intensity projections generated from the helical scan, respectively, with the fiducial marker and capsule surface highlighted. Although in the *ex vivo* setting the vascular network has collapsed and the blood has drained, we were still able to measure remnants of blood in vessels visible over the 360° field of view and up to a penetration depth of ~1.2 mm.

## 4 Discussion and conclusion

While holding tremendous promise for GI tract diagnostics, optoacoustic endoscopes have incomplete fields of view, low B scan frame rates, inappropriate probe construction and insufficient SNR for human esophageal imaging. We have enabled for the first time a 50 Hz, full 360° field of view optoacoustic capsule endoscope by integrating a side-looking broadband focused transducer electrically coupled with slip rings and a central aperture for coaxial illumination. We demonstrate imaging of 1700 × 2000 × 208 (ρ,φ,z) voxels, corresponding to a 0.24 Hz volume rate. We show imaging depths of 3 mm in low scattering media and an estimated imaging depth of ~0.84 mm for esophagus-mimicking phantoms at 100 kHz-laser repetition rates. The overall esophageal wall thickness in patients with Barrett’s and early cancer is 3–4 mm (including muscle layer) [25]. The thickness of Barrett esophagus has been measured by volumetric laser endomicroscopy and found to vary between 200 µm and 700 µm and it is generally always less than 900 µm [26]. Therefore, we believe that our OA endoscopy configuration should be able to image tissue within the depths expected from metastatic transformations occurring in Barrett’s esophagus.

Optoacoustic imaging was previously shown to reveal the vascular structure in skin melanoma and provide blood oxygenation maps to assess tumour angiogenesis and depth [14]. This performance could be useful for tracking esophageal lesions where the wall is rich in vasculature. Our group has recently proposed optoacoustic capsule endoscopy for radial imaging of the GI track [21]. This approach enabled uninterrupted 360° imaging for the first time, yet the B frame rate was limited to 2.5 Hz, preventing fast volumetric scanning. In the current work, we have achieved a 20-fold higher B scan frame rate by integrating the rotation coupling directly onto the transducer housing inside the capsule with customised miniature slip rings. To our knowledge, this enabled for the first time video-rate optoacoustic capsule endoscopy at 50 Hz which could lead to real time optoacoustic endoscopy surveillance of the GI track and can mitigate motion artefacts. We assessed the influence of pulse repetition rate (20–100 kHz) and B scan frame rate (10–50 Hz) on lateral resolution. Increasing the laser pulse repetition rate or the B scan frame leads to a reduced angular sampling resolution, which is expected from the increased interval between pulses available on each rotation cycle (number of A lines per revolution). We found that the maximum lateral resolution can be reached by sampling at 1/10th of the characteristic transducer lateral resolution at focus. Transducer characterisation yielded a 200 µm lateral resolution, leading to arc sampling of 20 µm required at focus. At 100 kHz-pulse repetition rate and 50 Hz-B frame rate, the arc sampling interval was 33 µm (φ=0.18°), which showed a degradation of the maximum lateral resolution to 234 µm, with an interquartile variation range of 16 µm. In contrast, at the same repetition rate, reducing the B scan frame rate to 30 Hz results in an arc sampling interval of 18 µm (φ=0.1°), improving the achievable resolution to 208 µm, closer to the maximum attainable value of the transducer. As expected, reducing the B scan frame rate further allowed a decrease down to 6.2 µm (φ=0.035°) at 10 Hz; however, this does not lead to a resolution improvement as it lies below the transducer limit.

We showcase the ability to acquire large volumetric imaging at 0.24 Hz over a total volume of 5mm×360°×10mm(ρ,φ,z)or 1790 mm^3^, at a high sampling resolution of 3μm×0.18°×48μm(ρ,φ,z). This high-resolution volumetric imaging speed can enable a complete scan of the entire human esophagus up to a length of 250 mm [27], in under 2 min. The ability to rapidly capture large volumes enables analysis of multiple high risk regions within the human GI tract including large Barrett’s esophagus segments. In media without scattering, the OA imaging penetration depth exceeded 3 mm. However, we show that the penetration depth exponentially depends on the scattering coefficient of the medium. The maximum transducer sensitivity at focus is preserved in low scattering cases ($\mu_{s}^{\prime }$≤0.049); however for higher scattering media, light diffusion becomes dominant over the transducer sensitivity response, shifting the maximum sensitivity towards the near-field of the transducer. Following the exponential relationship with increasing $\mu_{s}^{\prime }$, we estimate the imaging penetration depth in the human esophagus of our system to be 0.84 mm (assuming a $\mu_{s}^{\prime }$~1.01 @ 532 nm). We validated our phantom model by performing a helical pullback scan of an *ex vivo* pig esophagus at the same volumetric image rate of 0.24 Hz. Although the vascular network in the sample had collapsed, we were still able to measure remnants of vessels up to a depth of ~1.2 mm. We observed deeper structures in the pig esophagus than could be predicted from our phantom imaging (Fig. 4). The discrepancy can be attributed to the greater proportion of lower acoustic frequency components from the thick vessels in the pig esophagus, which experience lower propagation losses. For instance, the mesh in the phantom has a dimeter of 0.25 mm, whereas the blood vessels in the pig esophagus measured in Fig. 5(f) were ~0.7 mm in diameter. A second pig esophagus sample was scanned under the same conditions and revealed preserved vascular structures at imaging depth up to 0.96 mm (see Supplementary Fig. S1).

This depth is on par with previous OA studies, demonstrating images of small animal esophagus or colon tissue with penetration depths of ~ 1 mm in vivo at < 5 Hz [17], [18], [20]. As a result of the low frame rate coupled with low laser pulse repetition rates, the MPE rule 1 (20 mJ/cm^2^) is not violated (see supplementary). Under these conditions He et al. [21] were able to demonstrate an imaging penetration depth of 2 mm in pig esophagus ex vivo. Increasing the pulse repetition rates imposes tighter limitations on the MPE calculation where 20 mJ/cm^2^ is no longer an applicable limit. In our case, the MPE limit is 1.53 mJ/cm^2^ (see Supplementary). Previously, a 300 kHz pulse repetition rate with 30 µJ-pulse energies with a 1 mm grin lens producing a collimated beam were used in a catheter-based optoacoustic endoscope system [19]. The authors reported an energy density of 15 mJ/cm^2^, and following the limited aperture criterion of 3.5 mm of the ANSI standard, the MPE is exceeded by 174-fold in this case. These findings demonstrate that the translational potential of optoacoustic endoscopy is driven by the ability to rapidly scan the human esophagus, albeit currently exceeding the MPE by a factor of 4, while offering high resolution over several mm’s of depth.

The ex vivo performance of the optoacoustic capsule design presented herein represents a significant leap toward video-rate in vivo optoacoustic endoscopy of the human GI tract. For in vivo measurements, we anticipate that the capsule will stretch the esophageal track and thereby achieve optimal contact between the tissue surface and capsule by peristaltic force. More sophisticated balloon configurations could alternatively be explored. However, in order to translate the design for in vivo imaging, future efforts are required to address capsule size, sensitivity, resolution and motor stability compensation. While our current capsule design has a diameter of 16 mm, similar to standard WLSE, further miniaturization is required to ease swallowing and match the size of commercially available white light capsules, with sizes typically less than 32 mm in length and 13 mm in diameter [28], [29]. For instance, the overall length and diameter can be reduced by reducing the size of the transducer and by integrating a miniature motor inside the capsule. To further increase the sensitivity and imaging depth, a transducer pre-amplification stage can be integrated inside the capsule [21] while focusing the transducer sensitivity towards the surface of the capsule, thereby increasing the SNR for imaging in high scattering media. To increase the resolution of the optoacoustic images, we anticipate that a transducer with higher central frequency and reduced focal length, as currently employed in raster scanning optoacoustic mesoscopy, could be incorporated [10], [30]. Furthermore, image post-processing techniques can be explored to compensate for motor instability during scans. In this study only a single light source at 532 nm was utilized due to the lack of high repetition rate pulse lasers available at alternative wavelengths. To increase penetration depth by employing light sources in NIR region, where light attenuation in tissue is reduced, our group is investigating the possibility of over driving laser diodes of several wavelengths at hundreds of kilohertz and realizing their potential for video rate optoacoustic endoscopy [31]. Furthermore, oxygenation saturation measurements require illumination with at least two wavelengths, which would require wavelength multiplexing and reducing imaging speed by a factor equal to the number of wavelengths used. Alternatively, to retain high imaging speed, the spatial angular sampling rate can be reduced by a factor corresponding to the number of wavelengths used at the cost of image resolution. Our group recently investigated the use of nanosecond-pulsed laser diodes for OA imaging using frequency comb modulation to enable simultaneous laser wavelength illumination at high pulse repetition rates and enable oxygen saturation measurements without compromising imaging speed and quality [32].

In conclusion, a 16 mm optoacoustic capsule endoscope prototype with a side viewing central aperture endoscopic transducer with integrated optics and slips rings allowed for full field of view optoacoustic imaging at 50 Hz frame rate with angular sampling resolution of 0.18º attributed to the 100 kHz pulse repetition rate. Its functional potential was assessed in phantoms and an *ex vivo* pig esophagus where lateral resolution accuracy and imaging depth, respectively, were demonstrated. Overall, these experimental results suggest that the novel video-rate optoacoustic capsule endoscope design can mitigate motion artefacts during imaging, offering promising translational characteristics for human GI tract molecular imaging in the future.

## Declaration of Competing Interest

The authors declare no conflict of interest.

## References

[1] World Health Organization. International Agency for Research on Cancer. The Global Cancer Observatory. 2020. 〈https://gco.iarc.fr/today/fact-sheets-cancers〉 Accessed 21 Jan 2021.

[2] Visaggi P., Barberio B., Ghisa M., Ribolsi M., Savarino V., Fassan M., Valmasoni M., Marchi S., de Bortoli N., Savarino E. (2021). Modern diagnosis of early esophageal cancer: from blood biomarkers to advanced endoscopy and artificial intelligence. Cancers.

[3] Weusten B., Bisschops R., Coron E., Dinis-Ribeiro M., Dumonceau J.M., Esteban J.M., Hassan C., Pech O., Repici A., Bergman J., di Pietro M. (2017). Endoscopic management of Barrett’s esophagus: European society of gastrointestinal. Endosc. (ESGE) Position Statement Endosc..

[4] Bird-Lieberman E.L., Neves A.A., Lao-Sirieix P., O’Donovan M., Novelli M., Lovat L.B., Eng W.S., Mahal L.K., Brindle K.M., Fitzgerald R.C. (2012). Molecular imaging using fluorescent lectins permits rapid endoscopic identification of dysplasia in Barrett’s esophagus. Nat. Med.

[5] Menon S., Trudgill N. (2014). How commonly is upper gastrointestinal cancer missed at endoscopy? a meta-Analysis. Endosc. Int Open.

[6] Yoon J., Joseph J., Waterhouse D.J., Luthman A.S., Gordon G.S.D., di Pietro M., Januszewicz W., Fitzgerald R.C., Bohndiek S.E. (2019). A clinically translatable hyperspectral endoscopy (HySE) system for imaging the gastrointestinal tract. Nat. Commun..

[7] Attila T., Faigel D.O. (2009). Role of endoscopic ultrasound in superficial esophageal cancer. Dis. Esophagus.

[8] Xia F., Mao J., Ding J., Yang H. (2009). Observation of normal appearance and wall thickness of esophagus on CT images. Eur. J. Radiol..

[9] Kierszenbaum A.L., Tres L. (2011).

[10] Aguirre J., Schwarz M., Garzorz N., Omar M., Buehler A., Eyerich K., Ntziachristos V. (2017). Precision assessment of label-free psoriasis biomarkers with ultra-broadband optoacoustic mesoscopy. Nat. Biomed. Eng..

[11] Schwarz M., Soliman D., Omar M., Buehler A., Ovsepian S.V., Aguirre J., Ntziachristos V. (2017). Optoacoustic dermoscopy of the human skin: tuning excitation energy for optimal detection bandwidth with fast and deep imaging in vivo. IEEE Trans. Med. Imaging.

[12] Farnsworth R.H., Lackmann M., Achen M.G., Stacker S.A. (2014). Vascular remodeling in cancer. Oncogene.

[13] Haedicke K., Agemy L., Omar M., Berezhnoi A., Roberts S., Longo-Machado C., Skubal M., Nagar K., Hsu H.-T., Kim K., Reiner T., Coleman J., Ntziachristos V., Scherz A., Grimm J. (2020). High-resolution optoacoustic imaging of tissue responses to vascular-targeted therapies. Nat. Biomed. Eng..

[14] Omar M., Schwarz M., Soliman D., Symvoulidis P., Ntziachristos V. (2015). Pushing the optical imaging limits of cancer with multi-frequency-band raster-scan optoacoustic mesoscopy (RSOM). Neoplasia.

[15] Hong S.J., Kim T.J., Nam K.B., Lee I.S., Yang H.C., Cho S., Kim K., Jheon S., Lee K.W. (2014). New TNM staging system for esophageal cancer: what chest radiologists need to know. RadioGraphics.

[16] Ntziachristos V. (2010). Going deeper than microscopy: the optical imaging frontier in biology. Nat. Methods.

[17] Yang J.-M., Favazza C., Chen R., Yao J., Cai X., Maslov K., Zhou Q., Shung K.K., Wang L.V. (2012). Simultaneous functional photoacoustic and ultrasonic endoscopy of internal organs in vivo. Nat. Med..

[18] Li Y., Lin R., Liu C., Chen J., Liu H., Zheng R., Gong X., Song L. (2018). In vivo photoacoustic/ultrasonic dual-modality endoscopy with a miniaturized full field-of-view catheter. J. Biophotonics.

[19] Li Y., Zhu Z., Jing J., Chen J., Heidari A., He Y., Zhu J., Ma T., Yu M., Zhou Q., Chen Z. (2018). High-speed integrated endoscopic photoacoustic and ultrasound imaging system. IEEE J. Sel. Top. Quantum Electron. PP.

[20] Lei H., Johnson L.A., Eaton K.A., Liu S., Ni J., Wang X., Higgins P.D.R., Xu G. (2019). Characterizing intestinal strictures of Crohn’s disease in vivo by endoscopic photoacoustic imaging. Biomed. Opt. Express.

[21] He H., Stylogiannis A., Afshari P., Wiedemann T., Steiger K., Buehler A., Zakian C., Ntziachristos V. (2019). Capsule optoacoustic endoscopy for esophageal imaging. J. Biophotonics.

[22] M. Ferhatoglu, T. Kıvılcım, Anatomy of Esophagus, 2017.

[23] Michels R., Foschum F., Kienle A. (2008). Optical properties of fat emulsions. Opt. Express.

[24] Sweer J.A., Chen M.T., Salimian K.J., Battafarano R.J., Durr N.J. (2019). Wide-field optical property mapping and structured light imaging of the esophagus with spatial frequency domain imaging. J. Biophotonics.

[25] Gill K.R.S., Ghabril M.S., Jamil L.H., Al-Haddad M., Gross S.A., Achem S.R., Woodward T.A., Wallace M.B., Raimondo M., Hemminger L.L., Wolfsen H.C. (2010). Variation in Barrett’s esophageal wall thickness: is it associated with histology or segment length?. J. Clin. Gastroenterol..

[26] Levink I.J.M., Wolfsen H.C., Siersema P.D., Wallace M.B., Tearney G.J. (2019). Measuring Barrett’s epithelial thickness with volumetric laser endomicroscopy as a biomarker to guide treatment. Dig. Dis. Sci..

[27] B. Kuo, D. Urma, Esophagus - anatomy and development, GI Motility online 2006.

[28] Shamsudhin N., Zverev V.I., Keller H., Pane S., Egolf P.W., Nelson B.J., Tishin A.M. (2017). Magnetically guided capsule endoscopy. Med. Phys..

[29] Koulaouzidis A., Iakovidis D.K., Karargyris A., Rondonotti E. (2015). Wireless endoscopy in 2020: Will it still be a capsule?. World J. Gastroenterol..

[30] Ali Z., Zakian C., Ntziachristos V. (2021). Ultra-broadband axicon transducer for optoacoustic endoscopy. Sci. Rep..

[31] Stylogiannis A., Prade L., Buehler A., Aguirre J., Sergiadis G., Ntziachristos V. (2018). Continuous wave laser diodes enable fast optoacoustic imaging. Photoacoustics.

[32] A. Stylogiannis, L. Prade, S. Glasl, Q. Mustafa, C. Zakian, V. Ntziachristos, Frequency Comb Optoacoustic Tomography, bioRxiv, 2021, 2021.05.12.443808. 10.1038/s41467-022-32175-6 PMC9343396 35915111

