# Supplementary material for: 360º optoacoustic capsule endoscopy at 50 Hz for esophageal imaging
Source: Photoacoustics. 2022 Feb 1;25:100333. doi: 10.1016/j.pacs.2022.100333 (PMC8864533; doi:10.1016/j.pacs.2022.100333)
Supplement: Supplementary file 1 — Supplementary material [file mmc1.docx]

**Supplementary**

**Maximum permissible exposures for optoacoustic endoscopy**

The maximum permissible exposures (MPE) are a set of maximum laser energy values to which the eye or skin may be exposed without adverse effects or injury occurring in normal circumstances. The levels have been established in standard ANSI Z136.1 various wavelengths and exposure durations in the eye or skin. Due to a lack of scientific data on the laser safety level on esophageal tissue, we based our safety requirements on laser radiation of the human skin, with the important note that the MPE of esophageal tissue might be lower than that.

For repetitively pulsed lasers the MPE is always the smallest MPE value determined by the application of the ANSI Rules 1 and 2 [ ANSI Std. Z136.1-2000 (8.2.3.2)] applied over the area of the limiting aperture of 3.5 mm for skin imaging

$$MPE=min[MPE_{rule1},MPE_{rule2}]$$

Each of the MPE rules are defined as follows:

**MPE Rule 1 (Single Pulse):** No single pulse in a train of pulses shall exceed the MPE for a single pulse of the same pulse duration. For laser pulse duration of 1ns to 100 ns the single pulse radiant exposure over the wavelength of 400-700nm is 20 mJ/cm^2^. Our laser has a pulse duration of 1 ns with an operating wavelength of 532nm.

$$MPE_{rule1}=20\frac{mJ}{cm^{2}}$$

**MPE Rule 2 (Average Power):** The average MPE per pulse distributed over a number of laser pulses (N) over the exposure time(t) is given by:

$$MPE_{rule2}=\frac{MPE_{T}}{N}$$

Where N is the number of pulses during the exposure period given by the product of the pulse repetition rate(PRR) and exposure time (t) and the $MPE_{T}$ radiant exposure for an exposure time between 100 ns to 10 s over the wavelength range of 400-700nm from the ANSI skin safety table is given by:

$$MPE_{T}=1.1*{10}^{4}t^{0.25}J/m^{2}=1.1*t^{0.25}J/{cm}^{2}$$

Therefore the average MPE per pulse distribution as a function of PRR and exposure time t is given as follows:

$$MPE_{rule2}=\frac{1.1*t^{0.25}}{Int(PRR*t)}J/{cm}^{2}$$

Note: the number of pulses in an exposure is always considered as an integer for laser hazard evaluations.

The exposure time interrogating the same sample location is a function of the laser spot size at the capsule window interface, in our case we apply the limited aperture for skin imaging with a diameter of 3.5 mm. Rotating at the maximum speed of 50 Hz (314.15 rad/s) at 100kHz pulse repetition rate the exposure time t is given by

$$t=\frac{Interrogation spot central angle ( rad)}{frame rate (\frac{rad}{s})}=\frac{0.437}{314.15}=1.39ms$$

**Therefore:**

$$MPE_{rule2}=\frac{1.1*{1.39ms}^{0.25}}{Int\left( 100kHz*0.139ms \right)}=1.53\frac{mJ}{cm^{2}}$$

**Summary:**

Therefore, the maximum permissible limits for a pulsed laser beam interrogating tissue with a spot size of approximately 500 µm, central wavelength of 532 nm, pulse duration of 1 ns and repetition rate of 100 kHz is given by the following table:

| MPE rule 1 (mJ/cm^2^) | MPE rule 2(mJ/cm^2^) |
| --- | --- |
| 20 | 1.53 |

The minimum of the ANSI rules is MPE rule 2, yielding the MPE of our endoscope to be 1.53 mJ/cm^2^

During the course of all experimentation performed in this report an energy per pulse of approximately 12 µJ was employed. Hence energy density $E_{D}$ employed was:

$$E_{D}=\frac{0.012}{\pi*({0.025}^{2})}=6.11\frac{mJ}{cm^{2}}$$

Our density is $6.11mJ/cm^{2}$ and we thus exceed the maximum permissible energy of skin exposure by a factor of 4. To ensure this limit is not compromised, the repetition rate could be reduced from 100 kHz to 25 kHz to give a $MPE_{rule2}=$ 6.1$mJ/cm^{2}$ , although reducing repetition rate and keeping the frame rate fixed at 50 Hz corresponds to a lower lateral resolution as illustrated in Figure 2(e) at 50 Hz frame rate the lateral resolution is reduced from 234 $\mu$m at 100 kHz to 289$\mu$m at 20 kHz repetition rate. To ensure the sampling interval is not compromised during high speed imaging the SNR of our transducer requires further enhancement by at least a factor of 4.

**Helical volumetric scan of an ex vivo female pig esophagus sample**

**
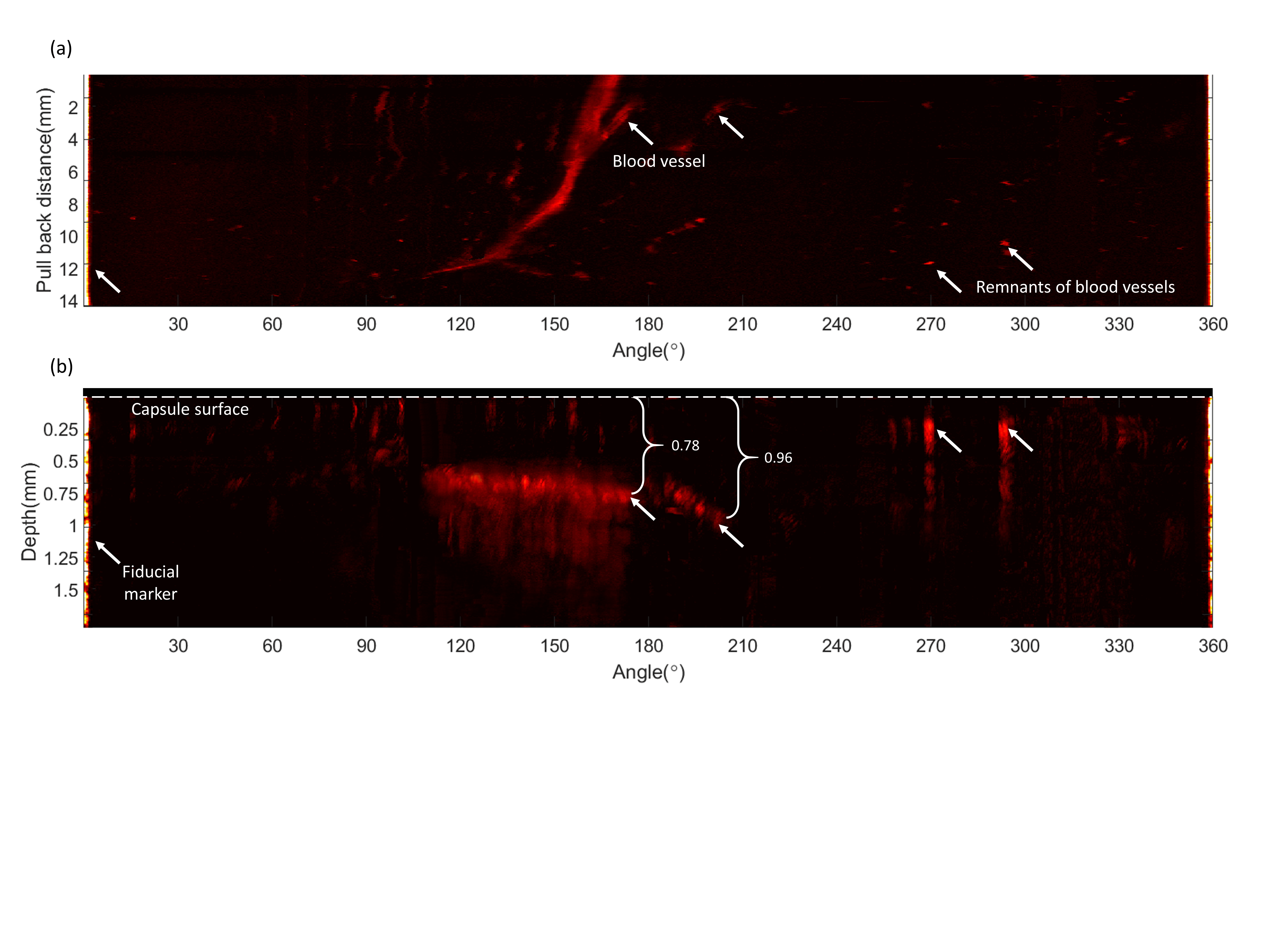
**

**Figure S1: 360º helical volumetric scanning of an ex vivo female pig esophagus sample over a 14 mm longitudinal pullback distance.** Maximum intensity projection for (a) enface and (b) side views.

**Comparison of reported human and pig oesophageal optical properties**

In our phantom model, we split the contribution of blood absorption using the mesh and the scattering contribution using intralipid. Authors in Ref [1] argue that the large variability reported is due to difference in the sample processing leading to high interpatient variability. Moreover, the standard deviation in Ref [2] was not given at 532 nm. Authors in Ref [3] argue that the large measurement variability for the reduced scattering coefficient reported is due to instrumental errors and sample inhomogeneity such as those introduced by narrow blood vessels, altering therefore the measurement. Moreover, it is possible that measurement variability can also results from unknown sampling depth in vivo. In Ref [4], the value for the reduced scattering coefficient is given as the mean of four measured layers of the esophagus, including the mucosa, submucosa, muscularis, and adventia, and are weighted by the layer thickness. The amalgamation of layers could explain the larger value reported, rather than that expected for the mucosal layer alone. We therefore chose a scattering coefficient reported for with the smallest standard deviation for superficial mucosal layers in Ref [5].

**Table S1: Reported esophagus optical properties**.

| Reference | Year | Wavelength  (nm) | $\mu_{s}^{'}$  $(mm^{-1})$ | g | $\mu_{s}$  $(mm^{-1})$ | $\mu_{a}$  $(mm^{-1})$ | Sample type |
| --- | --- | --- | --- | --- | --- | --- | --- |
| 3 | 1994 | 514 | 1.9 ± 1.3 | - | - | - | In vivo, human esophagus |
| 2 | 2007 | 532 | 0.82 ^§^ | 0.94 ^§^ | 13.6 ^*^ | 0.61^*^ | Ex vivo, human esophagus covering the entire mucosal depth |
| 1 | 2015 | 532 | 0.82 ± 0.48 ^§^ | 0.946 ± 0.028 | 15.2± 4.2 | - | Ex vivo, healthy human esophagus epithelium |
| 4 | 2018 | 514 | 2.84 ± 0.48 | 0.77 ± 0.04 | 11.83 ± 1.39 | 0.23 ± 0.03 | Ex vivo, pig esophagus in full, including muscle layers |
| **5** | **2019** | **526** | **1.01 ± 0.14** | **-** | **-** | **0.176 ± 0.042** | **Ex vivo, superficial human esopahgus** |

* Extracted from graph

§ Calculated

**Optoacoustic endoscopy precision golden slip rings design**

The endoscopic slip ring coupling configuration is depicted in figure S2. The wires maintain electrical connection while the rings spin. To minimize friction induced electrical noise during rotation, the rings are covered with a 5µm- thick gold layer. Two wires are in contact with each ring, making four V contact points per pole, thereby reducing the risk of losing the electrical contact during rotation. To further support electrical contact between the rings and wires, a brass casing around the joint ensures the wires are unable to stretch outwards. The brass enclosure is also grounded to the coaxial cable transmitting optoacoustic signals in an attempt to reduce electromagnetic interference.


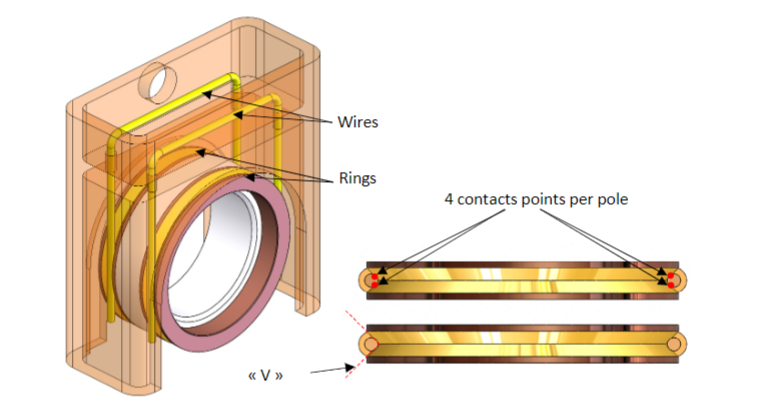


**Figure S2:Transducer slip ring coupling design**

**Optoacoustic endoscopy probe characterization procedure**

The optoacoustic endoscope transducer characterization configuration is illustrated Figure S3. The endoscope is mounted onto a 3-axis motorized linear stage XYZ (MTS50-Z8 in 3-axis XYZ Configuration Thorlabs) and immersed inside a 3D printed water tank with a 20 µm suture generating broadband optoacoustic signals. To determine the transducer’s characteristics, the linear motorized stages are used to linearly raster scan in the YZ-plane in step intervals of 5 µm in the z-axis and 10 µm in the y-axis, which encompasses the maximum envelope of each A-scan when obtaining the transducer’s sensitivity map. The global maxima from the sensitivity map determines the A-scan, which was obtained at the transducers focal length. The FFT and envelope of this A-scan represent the bandwidth and axial resolution at focus, respectively. The lateral resolution is determined from a line scan across the suture in the z-axis and the depth-of-focus is determined from a line scan moving along the y-axis with respect to the suture.


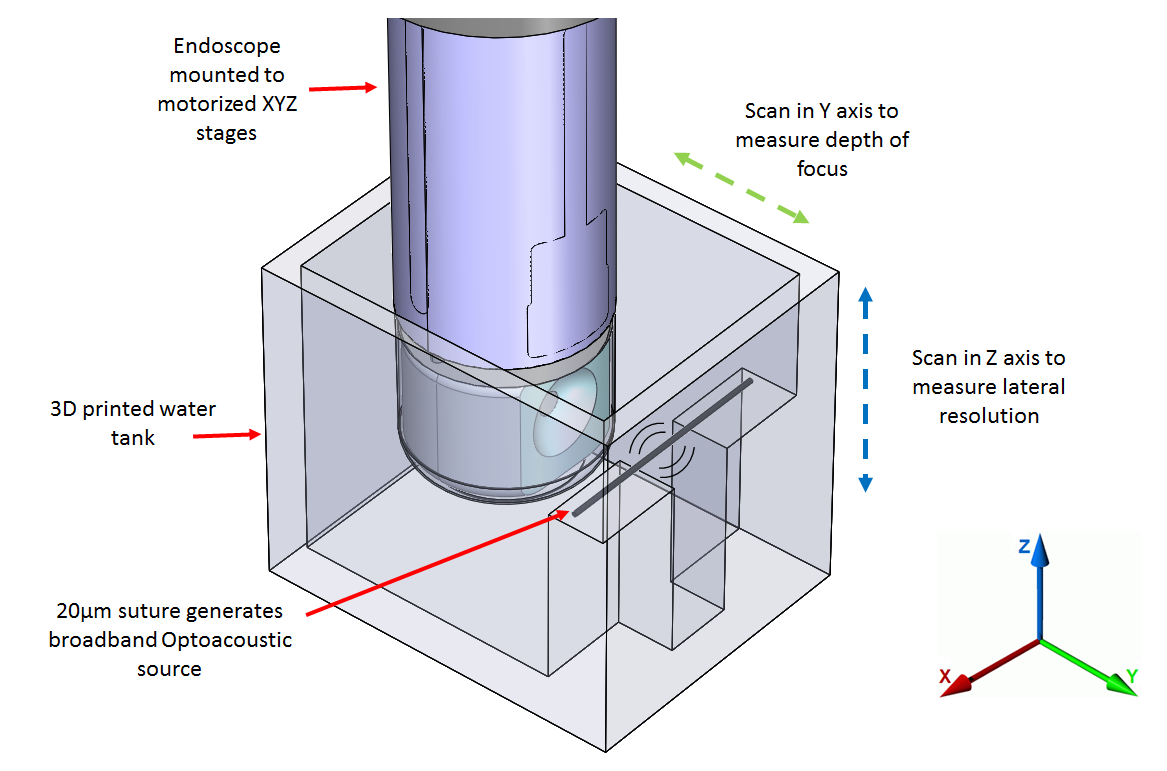


**Figure S3:optoacoustic endoscope transducer characterization setup**

**References**

1. J.-W. Su, Y.-H. Lin, C.-P. Chiang, J.-M. Lee, C.-M. Hsieh, M.-S. Hsieh, P.-W. Yang, C.-P. Wang, P.-H. Tseng, Y.-C. Lee, K.-B. Sung, Precancerous esophageal epithelia are associated with significantly increased scattering coefficients, Biomedical optics express 6(10) (2015) 3795-3805.
2. C. Holmer, K.S. Lehmann, J. Wanken, C. Reissfelder, A. Roggan, G. Mueller, H.J. Buhr, J.P. Ritz, Optical properties of adenocarcinoma and squamous cell carcinoma of the gastroesophageal junction, J Biomed Opt 12(1) (2007) 014025.
3. B. Roland, A.W. Georges, D. Robert, C.M. Jerome, B. Daniel, B. Hubert van den, S. Jean-Francois, M. Philippe, Clinical measurements of tissue optical properties in the esophagus, Proc.SPIE, 1994.
4. M. Hohmann, B. Lengenfelder, R. Kanawade, F. Klämpfl, A. Douplik, H. Albrecht, Measurement of optical properties of pig esophagus by using a modified spectrometer set-up, J Biophotonics 11(1) (2018).
5. J.A. Sweer, M.T. Chen, K.J. Salimian, R.J. Battafarano, N.J. Durr, Wide-field optical property mapping and structured light imaging of the esophagus with spatial frequency domain imaging, Journal of Biophotonics 12(9) (2019) e201900005
